# “What are you afraid of?” A mixed methods exploration of serious illness communication with oncology patients on general internal medicine wards in Canada

**DOI:** 10.1186/s12913-025-13512-z

**Published:** 2025-10-10

**Authors:** Isabelle Caven, Warren Lewin, Helen James, Amy Troup, Rajdeep Kaler, Leanne Kim, Senyo Agbeyaka, Aivan Chau, Richard Dunbar-Yaffe, Karen Okrainec

**Affiliations:** 1 University Health Network, Toronto, ON Canada; 2 Department of Family and Community Medicine, Division of Palliative Care, University of Toronto, Toronto, ON Canada; 3 Department of Supportive Care, Division of Palliative Care, University Health Network, Toronto, ON, Canada; 4 Department of Medicine, University of Toronto, Toronto, ON Canada; 5 Institute of Health Policy, Management and Evaluation, Dalla Lana School of Public Health, University of Toronto, Toronto, ON Canada; 6 Toronto General Hospital Research Institute, University Health Network, Toronto, ON Canada

**Keywords:** Serious illness communication, Advance care planning, Internal medicine

## Abstract

**Background:**

While emphasized as a key aspect of patient-centered care, little is known about the delivery and documentation of serious illness conversations (SIC) for patients with cancer admitted on general internal medicine (GIM) wards.

**Objectives:**

To characterize the documentation and experiences of SIC from the perspectives of patients and clinicians on GIM wards.

**Methods:**

This mixed methods quality improvement project gathered data from GIM wards using a: (1) retrospective review of electronic medical record data of hospitalized patients with cancer, (2) survey of physicians, residents, nurses, and allied health clinicians regarding their clinical practice, and (3) semi-structured interviews with hospitalized patients/caregivers.

**Results:**

The charts of 101 patients were reviewed: 85.2% had a documented code status, while less than half (46.5%) had documentation of a conversation with a clinician addressing hopes, concerns or values. Ninety-seven clinicians completed the survey and reported variable levels of documentation of SICs. Clinician-identified barriers to serious illness conversations included language barriers, prognostic uncertainty, and lack of time. The 21 patients/caregivers who were interviewed reported a lack of focus on values in their discussions with clinicians, and a desire for their clinician to tailor these discussions to their individual needs.

**Conclusion:**

In patients with cancer admitted to GIM wards, code status was frequently documented, whereas values-based components of these SICs were less frequently recorded Our results represent an opportunity to improve both the delivery and documentation of more holistic, person-centered aspects of serious illness conversations as a means to drive goal-concordant care through targeted clinical and educational interventions.

**Supplementary Information:**

The online version contains supplementary material available at 10.1186/s12913-025-13512-z.

Serious illness conversations (SICs) occur on a continuum addressing various aspects of patient/family illness understanding, prognostic awareness, hopes, values, and concerns to help patients live meaningfully and drive goal-concordant care [1, 2]. These components help clinicians move beyond having ‘code status’ discussions with their seriously ill patients to more holistic conversations that drives broad recommendations aligned to patient values, with code status being one such recommendation as part of some, and not all, discussions. Given the life-limiting nature of some cancers, and the potential for clinical deterioration during hospitalization, SICs are an essential part of inpatient care. Patients admitted for acute oncological care in Canadian academic hospitals are often cared for by general internists and trainees on General Internal Medicine (GIM) clinical teaching units (CTUs), in collaboration with allied health and nursing staff. Other specialties, such as oncology or palliative care, may act in a consultative role caring for patients with an oncological diagnosis, rather than leading patient care and SICs while admitted to hospital. Should a patient living with advanced cancer acutely deteriorate on a GIM ward during hospitalization, the default medical intervention in the absence of prior SIC documentation has traditionally been to attempt full resuscitation [3], which may not be clinically indicated nor aligned to patient/family preferences. At our institution, clinicians look at the documented code status (inclusive of cardiopulmonary resuscitation, intubation and mechanical ventilation) and/or for any pertinent SIC documentation, in the progress notes to direct in-the-moment conversations to guide treatments if time permits or to initiate treatment. Complicating this is that local and international data suggest that code status entry into a patient’s electronic medical record (EMR) occurs in approximately only 27% of hospital admissions [4] and 35–55% of cancer-specific admissions [5] leaving substantial room for improvement. Research has also identified a lack of clinician comfort and confidence in leading SICs [6–8]. Moreover, caregivers of cancer patients described distress or unmet communication needs surrounding prognostic information and end-of-life care with their medical team [9].

Across North America, various serious illness communication programs have been developed, implemented and evaluated, which have helped to improve the quality of SICs and move their focus beyond simply addressing code status; however, concerns about lack of time to have SICs and building relationships with patients have been identified as barriers to having fulsome discussions [10, 11]. At the academic teaching hospitals where this project took place, no widely adopted SIC interventions have been implemented and no standards for their evaluation exist. The implementation of a new EMR, which contains a specific section to document SICs, presented an important opportunity to evaluate documentation of SICs. Understanding the current state of SIC experiences from the perspectives of patients, families and all members of the healthcare team is an essential first step which served as a springboard for health systems interventions and quality improvement initiatives to leverage the SIC-related features of the new EMR and improve serious illness care at our institution. The following paper details the first phase of a quality improvement project, whereby an exploratory scan of the state of SICs on GIM wards was conducted (Fig. 1).The results subsequently served as the foundation for the second phase of the project (published separately), which was focused on co-designing interventions in clinical and educational practice [12, 13]. The objectives of this phase of our project were to: (1) determine the frequency in documentation of various components of SICs, (2) understand how clinician, contextual, and patient-specific factors influence the experiences and outcomes of SICs and their documentation into the EMR, and (3) explore the experiences of patients, families and clinicians who care for patients admitted to GIM wards with a diagnosis of cancer on SIC.

**Fig. 1:**
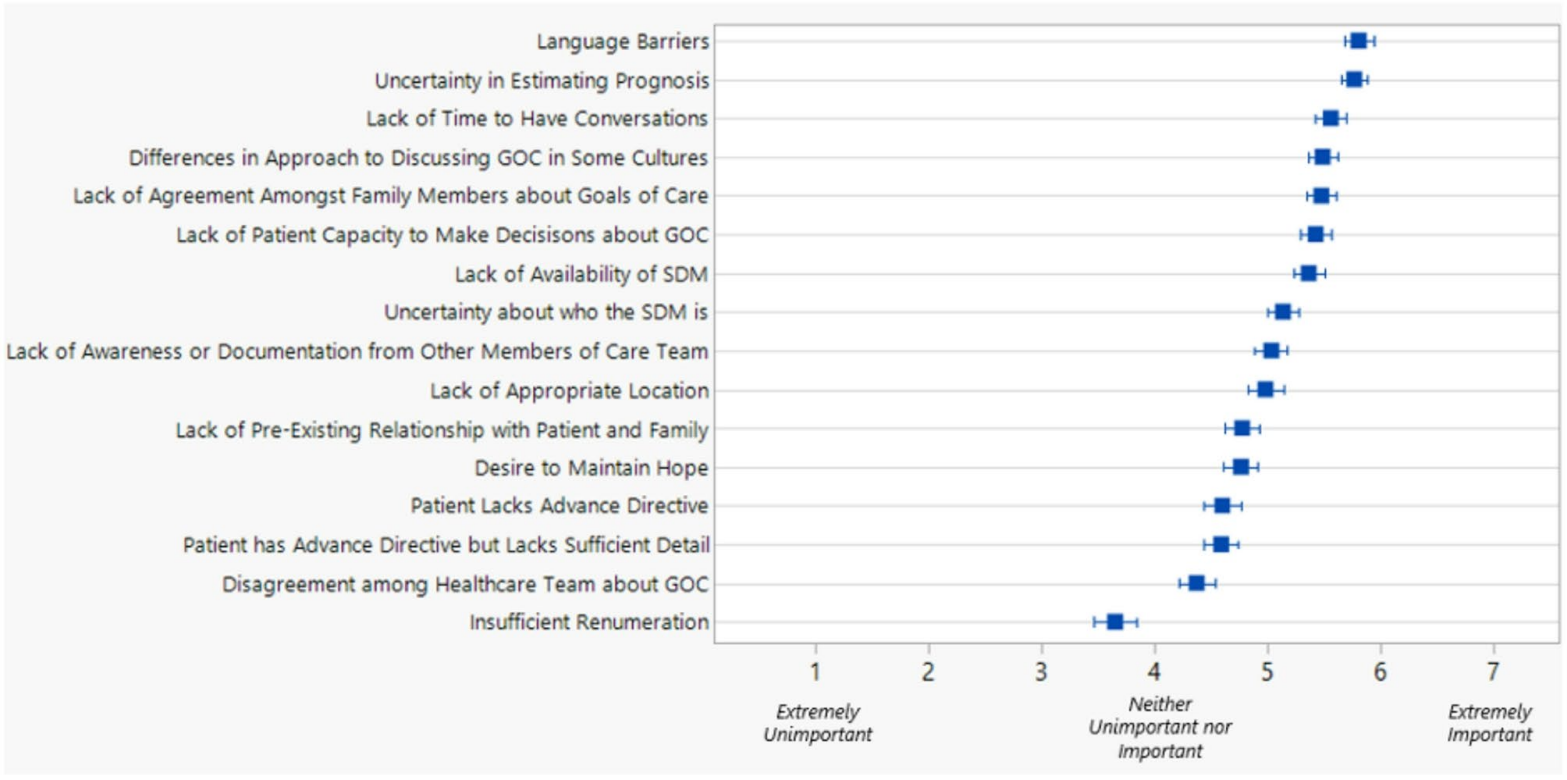
Mean ranks & standard error of clinicians’ perceived barriers to SICs

## Methods

### Study design

We used a convergent, parallel mixed methods design [14] which included: (1) a retrospective review of local EMR data, (2) cross-sectional survey of clinicians working on GIM wards, and (3) semi-structured interviews with patients and/or caregivers. Our team was led by two general internists, one a scientist in patient experience and communications research, another a clinician in quality improvement with leadership role as hospital site lead of GIM. Other team members included a palliative care physician, two residents (one specializing in palliative care, and one more junior), a nurse and a social worker, all of which work on the GIM units. For this project, SIC was defined to include code status, GOC and values-based discussions. At our institution, providers are able to specify details of patients wishes for resuscitation, such as CPR, intubation, and mechanical ventilation in the electronic health records. While code status may be discussed in isolation, decisions surrounding resuscitation may inform further conversations about treatment decisions based on patient values and are thus included in the definition of SICs. This project received Research Ethics Board exemption from the University Health Network and qualified as a quality improvement initiative. All standard ethical procedures were followed throughout the project.

### Participants

Eligible participants for a qualitative interview included patients admitted to GIM between January 2023 and March 2023 with an oncological diagnosis or their caregiver(s) when desired by the patient. The project employed convenience sampling, whereby after approval by the most responsible physician, patients/caregivers were approached by a member of the team at bedside or by telephone. Following discussion of the project goals and agreement to participate, the interview was conducted at bedside or post-discharge using Microsoft Teams. Additionally, patients/caregivers were recruited using flyers posted at the participating hospitals and instructed to contact the project team to participate. Eligible participants for the survey were GIM staff or resident physicians, nurses, physiotherapists, social workers, occupational therapists, dieticians, and physician assistants who work on GIM wards. All participants agreed to participate in the quality improvement project.

### Data collection

#### EMR chart review

The charts of 101 patients admitted to the GIM services at two academic hospitals between August 1, 2022 to March 5, 2023, with a primary oncological diagnosis or complication of one, were randomly selected and reviewed retrospectively. At our institution, 25–30% of total admissions to GIM wards are estimated to be patients with cancer, and across the seven-month chart review period, this would equate to 1000–1200, from which a sample of 101 was randomly drawn. Information related to diagnosis, code status, and elements of serious illness communication were abstracted, following the Serious Illness Conversation Guide created by Ariadne Labs [15] (See Appendix A for abstraction tool). Documented conversations that involved patient/caregiver hopes, worries, goals, fears or wishes about treatment, illness or life course were abstracted as values-based discussions. Two members of the project team independently completed the chart review and met to resolve discrepancies with project leads.

#### Clinician survey

Using REDCap [16], an online survey was distributed to clinicians who attend on the CTUs and GIM oncology units via email. SICs were defined broadly as “*any advance care planning*,* goals of care*,* family meeting*,* or code status conversation for an individual with cancer”*. Demographic information was documented. Respondents were also asked to rate the importance of barriers to conducting SICs, which were adapted from a survey on barriers to GOC discussions [17] (Appendix B). The original survey was developed via extensive literature review, focus groups, and consultations with providers across disciplines (general internal medicine, primary care, palliative care, critical care) to determine content and face validity. In the primary care setting, evidence of this questionnaire’s reliability and validity has been identified [18]. Respondents were asked to identify how often they took part in SICs with GIM patients who have an oncological diagnosis, how frequently they documented code status or values-based discussions, their comfort level with documentation, and at what point they believe code status should be documented in the EMR.

#### Qualitative interviews

Semi-structured interviews were conducted with patients admitted to GIM and their caregivers to explore their experiences with SICs. Following a review of the literature and discussion with the interdisciplinary project team, an interview guide was created (Appendix C). Interviews were led by two research assistants, who were trained in qualitative interviewing, and patient demographic information was collected. No participants were known to the research team prior to conducting the interview. All interviews were recorded, transcribed verbatim, and de-identified by members of the project team. Interviews were conducted until thematic saturation was reached.

### Data analysis

#### Quantitative analysis

Descriptive statistics were used to summarize participant characteristics from the chart review, clinician survey and interviews. To determine if there was a significant association between categorical variables from the chart review, χ2 tests were used. For the survey, respondents were asked to rank the importance of various barriers to engaging in SIC on a scale from 1 representing “Extremely Unimportant” to 7, representing “Extremely Important”.

#### Qualitative analysis

The transcripts from the qualitative interviews were independently coded by two members of the project team and thematically analyzed using direct content analysis [19], using NVivo v14. The transcripts were read line by line by two independent team members, who subsequently generated initial codes for the data. Initial codes were identified inductively, and themes were generated based on the identified codes. The codes and associated themes were reviewed with the interdisciplinary team.

#### Triangulation

As this work followed a convergent, parallel mixed methods design, the quantitative and qualitative strands were analyzed separately and then triangulated together to gain a more in-depth understanding of experiences with SICs. For example, documentation patterns, as identified through the EMR, were further contextualized through the clinician survey and patient interviews. The clinician survey explored both barriers to conducting SICs and comfort with documentation, allowing for comparison with EMR documentation patterns. Further, patient/family interviews provided important nuances to understanding how current conversations meet communication needs. The integration of qualitative and quantitative findings is further narratively explored in the discussion.

## Results

### Participant characteristics

The clinician survey was distributed to leaders of these groups which represented 220 residents, 40 staff physicians, 20 allied health clinicians and 330 nursing staff across the GIM sites. Ninety-seven respondents completed the survey in its entirety, resulting in a response rate of approximately 15.9%. Of the clinician participants, 55.6% were physicians (38 residents and 15 staff), 31.9% were nurses, and 12.3% were allied health (social work, occupational therapy, physical therapy, etc.) (Table 1). The charts of 101 patients were reviewed across the two sites. Throughout the interview period, 115 patients were screened for eligibility. Patients were not included in the interview if they declined to participate, were deemed not appropriate by staff for medical reasons, were discharged before contact or passed away. Twenty-one patients/caregivers were interviewed, most of whom were not admitted for a new cancer diagnosis but a complication of an existing cancer (90.5%).

**Table 1:** Demographic characteristics of survey and interview participants

	Total Clinician (*n* = 97)		Nurse (*n* = 31)		Allied Health (*n* = 12)		Physician (*n* = 54)		Patient/ Caregiver (*n* = 21)	
	*N*	%	*N*	%	*N*	%	*N*	%	*N*	%
**Site**										
Hospital A	56	57.7	21	67.7	8	66.7	27	50.0	18	85.7
Hospital B	41	42.3	10	32.3	*	*	27	50.0	*	*
**Gender**										
Female	64	66.0	23	74.2	12	100.0	29	53.7	10	47.6
Male	31	32.0	7	22.6			24	44.4	11	52.4
Prefer not to answer	*		*	*			*	1.9		
**Importance of Spirituality or Religion in life**										
Somewhat Unimportant/ Very Unimportant/ Extremely Unimportant	25	25.8	*	*	*	*	25	46.2	6	28.6
Somewhat Important/ Very Important/ Extremely Important	52	53.6	22	71.0	7	58.4	26	47.1	11	52.4
Prefer not to answer/ Not Applicable/ Neither Important nor Unimportant	20	20.6	*	*	*	*	*	*	*	*
**Racial/ethnic group** ^**1**^										
Other Race/Ethnicity	59	60.8	19	51.4	*	*	36	66.7	*	*
White	28	28.9	8	25.8	8	66.6	12	22.2	14	66.6
Prefer not to answer/ Do not know/ Other	10	10.3	*	*			6	11.1	*	*
**Preferred Language - English**	91	93.8	28	90.3	12	100.0	51	94.4	17	81.0
**Existing Cancer Diagnosis**									19	90.5
**Palliative Care Involvement during Admission**									3	14.3

*Results suppressed given < 5 responses. 1. Detailed racial/ethnic identities collapsed into categories given < 5 responses/category

#### Quantitative results- EMR data abstraction

Across the two hospitals, the frequency of code status entry and discussions of hopes, concerns or values with oncology patients admitted to the GIM services was 85.2% (*n* = 86) and 46.5% (*n* = 47) (respectively). Only 35.6% (*n* = 36) of these patients had a pre-existing code status documented in the EMR with the majority having code status documented as part of their SIC while admitted to hospital. The majority of documentation of SIC were by physicians, with a minority being documented by nurses, spiritual care or social workers. All patients who had a palliative care during their hospital admission (*n* = 32) had a values-based discussion. Of the 32 patients who engaged with palliative care during their stay, the majority (*n* = 28) had a values-based discussion with a palliative care provider. There was no significant association between patient language preference (English vs. Other) and code status entry in the chart (OR: 1.87, 95% CI 0.52 to 6.72) or between language and values documentation (OR: 0.64, 95% CI 0.23 to 1.79).

#### Quantitative results – clinician survey

A higher proportion of nurses (32.8%) and allied health (41.7%) than physician respondents (14.8%) reported having SICs more than 10 times a month (Table 2). Approximately a quarter (25.8%) of nurse respondents and 58.3% of allied health respondents reported feeling ‘Comfortable’ or ‘Very Comfortable’ conducting SICs. Almost all (92.6%) physician respondents reported feeling ‘Comfortable’ or ‘Very Comfortable’ conducting SICs. Nearly a quarter of the sample reported never or rarely documenting code status or values-based discussions (22.7%). The mean and standard error for barriers to SICs are presented in Fig. 1.

**Table 2:** Frequency of serious illness (SI) conversations and timing of code status Documentation

	Total (*n* = 97)		Nurse (*n* = 31)		Allied Health (*n* = 12)		Physician (*n* = 54)	
	*N*	%	*N*	%	*N*	%	*N*	%
**Frequency of SI Conversations**								
More than 10 times a month	23	23.7	10	32.3	5	41.7	8	14.8
**How often do you complete the following: documenting code status and/or conversation about values/wishes to guide treatment?**								
Never or Rarely	22	22.7	18	58.1	3	25.0	1	1.9
Sometimes or Often or Always	75	77.3	13	41.9	9	75.0	53	98.1
**How comfortable are you completing the following documents: code status order or documenting values and wishes?**								
Very Uncomfortable or Uncomfortable	12	12.4	9	29.0	1	8.3	2	3.7
Neutral	20	20.6	14	45.2	4	33.3	2	3.7
Very Comfortable or Comfortable	65	67.0	8	25.8	7	58.3	50	92.6
**When should code status be entered into Epic?**								
Pre-admission by Emergency Department	25	25.8	12	38.7	6	50.0	7	13.0
Immediately upon admission	42	43.3	12	38.7	3	25.0	27	50.0
After diagnostic clarification during admission	26	26.8	7	22.6	3	25.0	16	23.6
Adverse change in clinical trajectory	4	4.1					4	7.4

#### Qualitative results – patient/family interviews

Twenty-one patients and/or family members were interviewed, with interviews ranging in time between 7 and 50 min Three distinct themes were identified through qualitative analysis: (1) patient preferences for communication; (2) impact of care siloes; and (3) lack of values-based discussions. The themes and subthemes are described below and summarized in Table 3 with exemplary quotes.

**Table 3:** Qualitative themes and illustrative quotes

Theme	Subtheme	Illustrative Quotes
Patient Preferences for Communication	Varied experiences using the electronic patient portal	…. *alternatively it helped a little bit for her to prepare the questions that [patient] wanted to ask…it gave us a chance to mentally prepare what [patient] wanted to ask*,* she needs some time to process stuff. (Caregiver)* *But then at the same time*,* you go oh wait a minute*,* what does this mean? Who do I turn to*,* to find… like I’m anxious now because I think this says this… (Patient)*
	Desire for Tailored Communication	*understanding each person is going to respond in a different way… approaching them in a way that is almost personalized…(Patient)* *my own personality is to be quite direct… just give me the facts. (Patient)* *But I do think like if the doctor was a little bit more open in saying that*,* you know*,* what are your fears? Do you have any fears? I think it might make me as a person feel more open and comfortable to speak about them. (Patient)*
	Information in Multiple Formats	*For me the piece that would have been more helpful is maybe having some documentation*,* especially that I’m here through this experience predominately on my own. So at time*,* the stress makes it hard to remember things accurately (Patient)* …*maybe written stuff but also somebody that’s actually there to speak to you about it. (Patient)*
Impact of Care Silos	Rotating Staff	*I can’t develop this relationship because each time the doctor is changing (Patient)* *… we saw six different residents*,* we saw two different nurse practitioners and they often ask the same questions. So*,* it just seemed like a waste of their time cause the questions were already asked and I recognize that it is a teaching hospital um but at the end of the day…we know the mental well-being of the patient has a huge impact and it really did wear down our sense of trust and whether or not*,* really the care was*,* we’re just another number*,* another case. (Caregiver)*
Lack of Values-Based Discussions		*I don’t think anyone has ever talked about values. More have been like; this is the plan. (Caregiver)* *No that was just… they wanted to know whether or not I wanted a do not resuscitate order. And I said no*,* I’m not ready for that yet. (Patient)* *I had a healthcare provider once start off a conversation with me*,* how are you? What are you afraid of? I had never had a healthcare provider in an acute situation ask that. Which I thought was pretty fantastic (Patient)* *Interviewer: Would you have preferred them [healthcare team] to have initiated it [values-based discussions]?* *Patient: Maybe a part of me would have. I think it would have made me feel like I’m allowed to be vulnerable in that space. And um that*,* you know*,* talking about emotions is acceptable instead of only being allowed by biology and science and what’s actually going on physically instead of emotionally….*

##### Patient preferences for communication

Patients/caregivers described individual preferences for communication related to diagnosis, treatment, or illness progression. These preferences are organized under three categories: (1) experiences using the patient portal, (2) tailored communication, and (3) sharing information in multiple formats. For the first category, patients receiving care at this institution as inpatients and through outpatient clinics have access to their health information through an electronic health record, specifically, an online portal system, hereafter referred to as the patient portal, which includes documented notes from clinicians and lab results. Patients and caregivers expressed both positive and negative experiences with the patient portal. For some, having information accessible in real-time about their illness over the patient portal was an overwhelming experience:

*Maybe they can post the results later after we’ve spoken to a doctor. Um… as much as I would still be scared or whatever I would still feel like at least I’ve spoken to somebody*,* and seeing the results isn’t as scary anymore. (Patient)*

This patient also expressed a desire to have more context around the results shared in the patient portal, which was echoed by other patients. Another patient suggested a pop-up banner to accompany potentially distressing news explaining that a physician would contact them to explain the findings. Comparatively, other patients and caregivers described using the portal as empowering – having access to their health information gave them time to process the results and any associated emotions.

*I also think that alternatively it helped a little bit for her to prepare the questions that [patient] wanted to ask…it gave us a chance to mentally prepare what [patient] wanted to ask*,* she needs some time to process stuff. (Caregiver)*

The second communication preference identified was the use of tailored communication. Whereas some patients spoke about desiring their clinician to communicate with increased empathy during SICs, others preferred a more facts-based, direct approach.

*….part of my own sort of personality is to be quite direct*,* and you know*,* the facts only*,* just give me the facts*,* don’t try to smooth it over or make me feel better about it anything*,* or make me feel good about anything*,* just give me the facts*,* I know how to deal with it. (Patient)*

Patients spoke about wanting their clinician to understand their individual communication style and tailor their approach to it. The last communication preference identified by patients was having information communicated to them in multiple formats.

*They’ve [care team] been fantastic in answering my questions*,* you know*,* providing the information that I required in multiple formats…if you’re in front of them and they are verbally telling you*,* there’s a resource that you can go back and check if you’ve forgotten. (Patient)*

Having information delivered in multiple formats helped patients process and remember what their care team shared with them.

##### Impact of care siloes

While the interview was focused on serious illness conversations, many patients spoke about siloed care delivery, and how that impacted relationships with their clinicians. Some patients described challenges in coordinating care between their inpatient and outpatient clinicians affected by SIC. Others described the impact of frequent staff turnover in the academic teaching hospital:

I can’t develop this relationship because each time the Doctor is changing (Patient)

Additionally, some interviewees identified a lack of communication between their different clinicians and its impact on serious illness communication.

*I can say that kind of in passing I was told by a doctor that I had a heart attack and that was it. And then he kind of walked out of the room. … It was very clear he was just referencing something that he assumed was already discussed with me. But*,* when you’ve had multiple care providers*,* all in and out doing different things*,* it’s… how can you coordinate*,* you know*,* the flow of information? (Patient)*

##### Lack of values-based discussions

If patients described serious illness conversations, it was often in relation to do not resuscitate orders. Most patients/caregivers did not report discussing values relating to their care with their clinician.

*Interviewer: … when you were in the hospital*,* do you remember having these serious illness conversations about*,* you know*,* CPR*,* advanced care planning*,* anything like that with – [patient shakes head] Nobody?*

*Caregiver: Nope. Not once*,* no.*

This caregiver then described various points at their care journey where they felt it would have been appropriate for the care team to broach this topic but did not. When probed about values-based discussions, some patients described not feeling ready to have these conversations with their clinicians or not feeling they were necessary during their admission.

*It’s always been about the specific treatment*,* you know*,* what’s going on at the time*,* it’s always medical… But then as I said I’m just coming up to the point now where they are saying uh you might not do so well this time. You know? And so*,* whether they’ve got things planned down the road*,* I don’t know. To this point I’ve had none of it. I don’t really need any of it right now. But*,* it’s a good idea. (Patient)*

Alternatively, other patients expressed a desire for values-based discussions but had to initiate these with their clinicians.

*Interviewer: What is important to you in terms of your care? Have you had any of those conversations with anyone?*

*Patient: Actually*,* I was the one proposing that…. when they announced I might have ovarian cancer*,* for them it’s going to automatically be - ok we’re going to remove everything. And I was like*,* and if I don’t want to? Like*,* let’s say for example I want to have babies or something*,* what’s going to happen in this case? …. I can see their face like*,* it’s like it’s not an option for you. For me*,* that was ah… that was devastating because if I’m not asking that for myself*,* they probably not going to care that much for you.*

Another patient expressed a desire for conversations with their care team that addressed more than medical concerns (Table 3) and allowed them to bring their emotions and vulnerabilities into the conversation.

## Discussion

This mixed methods project presents several important results across a diverse group of participants and provides a foundation from which to implement targeted solutions to improve SICs on GIM wards. Despite a relatively high frequency of multidisciplinary providers who self-reported having SICs with patients admitted to GIM, documentation and comfort levels surrounding this type of communication were varied. The highest level of documentation of SICs was focused on code status discussions which had not been documented prior to admission. In contrast, there was a paucity of values-based discussions documented. The most important barriers to SICs for clinicians were language barriers, prognostication challenges, and limited time to have conversations. Findings from the patient/caregiver interviews aligned with the EMR review, such that code status discussions were commonplace, with limited exploration of individual values. Different aspects of the patient experience, such as care siloes, were also described as influencing serious illness conversations with their clinicians.

Our findings diverge with recent studies that identify relatively low rates of documentation of code status [5]; however, align with studies that identify low rates of GOC documentation [20]. Clinician survey respondents reported conducting SICs somewhat frequently; however, there was variability in the frequency of documentation amongst clinical roles. In the clinical context where this project took place, oncology patients admitted to GIM are cared for by general internists, resident physicians, nursing staff, and allied health. As needed, other physicians consult on patient care (i.e., oncology, palliative care). Variability in documentation both prior to admission by the treating oncologist and during admission by any clinical member of the health care team may in part be due to perceptions that serious illness conversations are the physician’s responsibility and do not need to be documented by other clinical staff. Previous work has demonstrated that hospital-based clinical staff view physicians as the most acceptable group to have SICs but identified important roles for allied heath staff in these discussions, such as initiating the conversation or acting as a decision coach [17]. In settings where serious illness interventions were implemented, supporting social workers and nurses to initiate SICs was seen as helping to move accountability for conversations taking place from the individual to the team [7]. Comfort with SICs, which was lower amongst the nursing and allied health respondents in the sample, may be improved by tailored SIC interventions. An adaptation of Ariadne Lab’s Serious Illness Care Program for nursing and allied health staff has been created, with a focus on clarifying scope of practice for communicating prognostic information [21]. While this resource has not yet been evaluated, it may be a useful tool in supporting nurses and allied health staff, who are already having SICs, in feeling empowered to do so as part of the clinical team. Our findings can further support the targeted implementation of this resources on GIM wards.

The barriers to SICs as perceived by clinician survey respondents have also been identified elsewhere to include lack of time [17, 22, 23], prognostication challenges [24], limited patient capacity [17, 25], language barriers and cultural differences [17]. Sufficient time is needed to explore the continuum of SICs with patients, with experts suggesting doing so at time of diagnosis can help downstream, including reducing the need for hospitalization [26]. Initiatives that look to improve lower rates of values-based GOC documentation should be prioritized alongside system-level barriers. For example, prognostication was identified in this work as a barrier to SICs by clinicians and was identified in another study as an important area of communication for caregivers of patients with serious illness and at end of life [9]. Prognostic information, once known, could be documented in the EMR prior to admission by oncologists, palliative care physicians, or other members of the outpatient care team to support inpatient clinicians in having the necessary details to conduct SICs.

Previous authors have also found reported discordance between patient and clinician reports of SICs, suggesting a potential lack of shared understanding as to what that discussion entails [20]. Our EMR chart review identified that there were relatively low rates of documentation of values-based discussions, and, importantly, contextualized by interviews where patients rarely described having in-depth SICs that centered on values, wishes or fears. These patients/caregivers may not have had SICs or may also have lacked a shared understanding in what SIC entailed [20]. This is an important finding as our organization supports clinicians to move away from asking patients what treatments they desire towards making clinical recommendations, based on training, the evidence, and experience, and importantly, aligned to patient values. Our patient interviews identified how information on SIC could be provided in multiple formats, through electronic patient portals, or pamphlets, to help prepare patients for a SIC and what it entails, and also support patients and families post-discharge to reflect on their inpatient SICs [22].

Our work has several important implications for in-hospital SICs and documentation and provides a necessary foundation to co-design interventions to address gaps in serious illness care on GIM wards. The growth of EMRs across hospitals at our site and internationally provides an opportunity for multidisciplinary teams to review the documentation of SICs in their institution, the variability in SICs, and to use the data as further springboards for quality improvement and educational interventions. At our site, these findings are being used by a multidisciplinary team of oncologists, palliative care, internal medicine clinicians to set quality standards and a common language for which co-designed interventions can be built on. In the context of serious illness, the patient-facing portal could be leveraged to (1) improve visibility of SIC documentation for patients to address patient/clinician discordance in regard to a SIC taking place; and (2) increase understanding of what SICs encompass by providing informational resources, including language and culturally concordant resources. Working with patients and clinicians to address actionable ways to improve SICs and documentation is an important next step to addressing the multi-faceted barriers to leading high quality SICs.

This quality improvement project has important limitations to address. First, while there was a large proportion of allied health professionals who reported having SICs, this sample size was relatively smaller than the other types of clinicians who completed the survey (physicians, nurses) and may not be reflective of the experiences of this group. Further, most patients/caregivers interviewed were white and identified English as their preferred language. Recent census data estimates approximately 24.2% of people living in the city where this project took place speak a language other than English or French as their mother tongue [27], therefore our sample of mostly English-speaking patients/caregivers is not representative of the diversity of languages. Previous work has identified that patients with limited English proficiency (LEP), or those from diverse cultural/racial backgrounds, experience barriers to SICs [28, 29]. Clinicians in this survey also identified language discordance as a barrier to SICs, but this was not explored with the sample of patients/caregivers interviewed. Patients with LEP are more likely to receive aggressive end-of-life care and less likely to complete an advance directive [30]. Culturally concordant care must be integrated into SICs, and future research of the perspectives and experiences of these patients/caregivers is needed to equitably improve SICs.

In conclusion, the findings from this quality improvement project demonstrate that code status documentation for oncology patients is high on internal medicine wards; however, values-based discussions, as identified both through review of EMR documentation and patient/caregiver interviews, are less frequent. SICs have been highlighted as an integral aspect of high quality, patient-centered cancer care, and the findings from this project demonstrate opportunities to move the needle of SICs beyond solely code status documentation at institutions such as ours where multidisciplinary teams support medical inpatients with underlying cancer diagnoses. Both individual (i.e., language) and clinician/system-level (i.e., prognostic uncertainty, lack of time) barriers were identified by clinicians as impacting SICs. Patients/caregivers that were interviewed described SICs that were not values-based and shared several key insights for how to improve these conversations to meet their personal preferences and communication needs. Despite being a central tenet of care delivery, communication skills training has not always been a central focus of clinician training. Next steps include working in collaboration with patients and all members of the clinical team to break down care silos, improve documentation of SICs while increasing the frequency of the values-centered aspects of SICs.

## Supplementary Information

Below is the link to the electronic supplementary material.

Supplementary Material 1

Supplementary Material 2

Supplementary Material 3

Supplementary Material 4

## Data Availability

The datasets used and/or analysed during the current study are available from the corresponding author on reasonable request.
