# Supplementary material for: “What are you afraid of?” A mixed methods exploration of serious illness communication with oncology patients on general internal medicine wards in Canada
Source: BMC Health Serv Res. 2025 Oct 10;25:1348. doi: 10.1186/s12913-025-13512-z (PMC12512328; doi:10.1186/s12913-025-13512-z)
Supplement: Supplementary file 1 — Supplementary Material 1 [file 12913_2025_13512_MOESM1_ESM.docx]

**EMR Data Abstraction Tool**

| **Medical Record Number (MRN)** |  |
| --- | --- |
| **Age** |  |
| **Sex** |  |
| **Gender Identity** | - Female - Male - Intersex - Trans Female-Male - Trans Male-Female - Unknown |
| **Preferred Language** | - Arabic - Bengali - Chinese (Cantonese) - Chinese (Mandarin) - English - Farsi - French - Hindi - Italian - Korean - Polish - Portuguese - Punjabi - Russian - Spanish - Tamil - Turkish - Urdu - Vietnamese - Other (specify) - Prefer not to answer |
| **Religion** | - Roman Catholic - Protestant Christian - Orthodox Christian - Other Christian - Muslim - Jewish - Buddhist - Hindu - Sikh - No religious affiliation - Other (specify): ___________________________ - Prefer not to answer |
| **Race** | - Asian – East (e.g., Chinese, Japanese, Korean) - Asian – South (e.g., Indian, Pakistani, Sri Lankan - Asian – South East (e.g., Malaysian, Filipino, Vietnamese) - Black – African (e.g., Ghanaian, Kenyan, Somali) - Black – Caribbean (e.g., Barbadian, Jamaican) - Black – North American (e.g., Canadian, American) - First Nations - Indian – Caribbean (e.g., Guyanese with origins in India) - Indigenous/Aboriginal – not included elsewhere - Inuit - Latin American (e.g., Argentinean, Chilean, Salvadoran) - Métis - Middle Eastern (e.g., Egyptian, Iranian, Lebanese) - White – European (e.g., English, Italian, Portuguese, Russian) - White – North American (e.g., Canadian, American) - Mixed heritage (e.g., Black – African and White – North American) (Please specify): …___________________________ - Other(s) (Please specify): …___________________________   Prefer not to answer |
| **Admission Date** |  |
| **Admitting Team** |  |
| **Most Responsible Physician (MRP)** |  |
| **Type of Provider documenting GOC or serious illness conversations**  **(Check all that apply)** | - Physician - Nurse - Social worker - Trainee - Other: _______________ |
| **Speciality of Provider documenting GOC or serious illness conversations**  **(Check all that apply)** | - GIM - Oncology - Palliative care - ICU - Other: ______________ |
| **Hospital Site** | - Hospital A - Hospital B |
| **Type of GIM service** | - Clinical Teaching Unit - Oncology- GIM unit - Other unit (e.g. ED, COVID unit): ____________ |
| **Oncological Care (if applicable) being Delivered at Attached Oncology Hospital or Another Hospital** | - Yes - No |
| **Admitting Diagnosis** |  |
| **Prognosis** | - <1 month - 1-3 months - 3-6 months - 6-12 months - > 12 months - Unknown/Not Found |
| **Documentation of Cancer Diagnosis and Type** | - Yes: - No |
| **Cancer Stage** |  |
| **Date of Cancer Diagnosis** | - Date: - Unknown - N/A |
| **New Cancer Diagnosis** | - Yes - No - N/A |
| **Expected Complications** |  |
| **Outpatient Cancer Care Provider** |  |
| **Who Documented GOC Discussions** |  |
| **Documentation of Patient Values (eg. to be independent, to eat, pain control, to be comfortable, getting to a milestone such as the birth of a grandchild…)** |  |
| **Resuscitation Wishes** |  |
| **Specific Recommendations from Provider documenting GOC discussion** |  |
| **Family Meeting needed for GOC discussions** | Yes Days after admission:  No |
| **Performance Status**  **(Palliative Performance Scale (PPS) or Eastern Cooperative Oncology Group (ECOG))** | - PPS:   *(Options are 0, 10, 20, 30 etc., 100%, not documented)*   - ECOG:   *(Options are 0-5, not documented)* |
| **Comorbidities/ Charlson Comorbidity Index (CCI)** |  |
| **Total Length of Stay (in days)** |  |
| **Acute Length of Stay (in days)** |  |
| **Pre-Existing Code Status** | - Yes - No - Not documented |
| **Code Status Discussion** | - Initiated - Completed - Not entered - Entered during previous admission (if yes, how many months ago) |
| **Palliative Care Involvement** | - Yes (pre-admission involvement) - Yes (peri-admission involvement) - No |
| **Discharge Destination** | - Home - Long term care/ nursing home - Rehab - Retirement home - Other hospital - Palliative care unit/ hospice - Died in hospital   Other: |
