# Supplementary material for: “What are you afraid of?” A mixed methods exploration of serious illness communication with oncology patients on general internal medicine wards in Canada
Source: BMC Health Serv Res. 2025 Oct 10;25:1348. doi: 10.1186/s12913-025-13512-z (PMC12512328; doi:10.1186/s12913-025-13512-z)
Supplement: Supplementary file 2 — Supplementary Material 2 [file 12913_2025_13512_MOESM2_ESM.docx]

**Demographic Questionnaire**

| ***OBTAINED AT TIME OF CONSENT*** | |
| --- | --- |
| **MRN and/or Participant ID:**  **Age (in years):**  **Birth year:**  **Site:**   - **TGH** - **TWH** | **Date of Consent:**  (DD-MMM-YY)  **Date of Baseline Data Collection:**  (DD-MMM-YY)  **Consent By:**   - Health Care Provider - Trainee - Patient - Caregiver   Relationship to Patient:  **Consent Obtained By:** |
| **Sex:**   - Female - Male - Prefer not to answer | **Gender:**   - Female - Male - Intersex - Trans – Female to Male - Trans – Male to Female - Other (specify): ___________________________ - Prefer not to answer |
| **Preferred Language:**   - Arabic - Bengali - Chinese (Cantonese) - Chinese (Mandarin) - English - Farsi - French - Hindi - Italian - Korean - Polish - Portuguese - Punjabi - Russian - Spanish - Tamil - Turkish - Urdu - Vietnamese - Other (specify): ___________________________ - Prefer not to answer - Do not know | **Which of the following best describes your racial or ethnic group? Check ONE only:**   - Asian – East (e.g., Chinese, Japanese, Korean) - Asian – South (e.g., Indian, Pakistani, Sri Lankan - Asian – South East (e.g., Malaysian, Filipino, Vietnamese) - Black – African (e.g., Ghanaian, Kenyan, Somali) - Black – Caribbean (e.g., Barbadian, Jamaican) - Black – North American (e.g., Canadian, American) - First Nations - Indian – Caribbean (e.g., Guyanese with origins in India) - Indigenous/Aboriginal – not included elsewhere - Inuit - Latin American (e.g., Argentinean, Chilean, Salvadoran) - Métis - Middle Eastern (e.g., Egyptian, Iranian, Lebanese) - White – European (e.g., English, Italian, Portuguese, Russian) - White – North American (e.g., Canadian, American) - Mixed heritage (e.g., Black – African and White – North American) (specify): ___________________________ - Other(s) (specify): ___________________________ - Prefer not to answer - Do not know |
| **Highest Level of Education Completed:**   - No Education - Less than High School - High School - Post-secondary Education (i.e., undergraduate studies) - Trade/Diploma/Certificate - Graduate Studies - Post-graduate Studies - Other (specify):   ___________________________   - Prefer not to answer - Do not know | **What is your religious or spiritual affiliation? Check *one* only.**   - Christian Orthodox - Protestant - Roman Catholic - Christian, *not included elsewhere on this list* - I do not have a religious or spiritual affiliation - Animism or Shamanism - Atheism - Baha’i Faith - Buddhism - Confucianism - Hinduism - Jainism - Judaism - Islam - Native Spirituality - Rastafarianism - Sikhism - Spiritual - Unitarianism - Pagan - Zoroastrianism - Other (specify): ______________________ - Prefer not to answer - Do not know |
| **How important is spirituality or religion in your life:**   - Extremely important - Very important - Somewhat important - Neither important nor unimportant - Somewhat unimportant - Very unimportant - Extremely unimportant - Not applicable - Prefer not to answer - Do not know |  |
| ***PATIENTS/CAREGIVERS ONLY*** | |
| **Type of Cancer:** | **Date of Diagnosis:** |
| ***RESIDENTS ONLY*** | |
| **What is your Current Position:**   - PGY1 - PGY-2 - PGY-3 - PGY-4 or above | **How many weeks of a Clinical Teaching Unit rotation have you completed so far during your residency?** ______ weeks |
| **Where did you graduate from Medical School?**   - Canada - USA - UK/Ireland/Australia/New Zealand - Europe - Asia - Middle East - Central or South America - Africa - Other (specify): ___________________________ - Prefer not to answer | **How often do you take part in serious illness conversations with patients admitted to the General Internal Medicine ward with primary oncological diagnosis or complications of one?**   - > 15 times a month - 10-15 times a month - 5-10 times a month - < 5 times a month - None |
| **Have you ever worked on a formal palliative care consultation or inpatient services?**   - Yes - No | **Have you had personal experience with a** **goals of care or serious illness conversation for a hospitalized close family member or friend?**   - Yes - No - Prefer not to answer |
| ***STAFF PHYSICIANS ONLY*** | |
| **Years in Practice:** | **Occupation:** |
| **How often do you take part in serious illness conversations with patients admitted to the General Internal Medicine ward with primary oncological diagnosis or complications of one?**   - > 15 times a month - 10-15 times a month - 5-10 times a month - < 5 times a month - None | **Have you ever worked on a formal palliative care consultation or inpatient services?**   - Yes - No |
| **Have you had personal experience with a close family member or friend being hospitalized in an intensive care unit?**   - Yes - No - Prefer not to answer |  |
| ***ALLIED HEALTH ONLY*** | |
| **Years in Practice:** | **Occupation:** |
| **How often do you take part in serious illness conversations with patients admitted to the General Internal Medicine ward with primary oncological diagnosis or complications of one?**   - > 15 times a month - 10-15 times a month - 5-10 times a month - < 5 times a month - None | **Have you ever worked on a formal palliative care consultation or inpatient services?**   - Yes - No |
| **Have you had personal experience with a close family member or friend being hospitalized in an intensive care unit?**   - Yes - No - Prefer not to answer |  |
