# Supplementary material for: “What are you afraid of?” A mixed methods exploration of serious illness communication with oncology patients on general internal medicine wards in Canada
Source: BMC Health Serv Res. 2025 Oct 10;25:1348. doi: 10.1186/s12913-025-13512-z (PMC12512328; doi:10.1186/s12913-025-13512-z)
Supplement: Supplementary file 3 — Supplementary Material 3 [file 12913_2025_13512_MOESM3_ESM.docx]

**Semi-structured Interview Guide**

**Participants: Patients and Caregivers**

Thank you for agreeing to participate in this research study. We appreciate you taking the time to share your experiences with us. The purpose of our study is to better understand the patient and family perspective on serious illness conversations and goals of care discussions.

***When we speak of “serious illness conversations”, we mean conversations with your health-care team which may have involved breaking bad news, advanced care planning, discussing your goals of care, resuscitation or other parts of your care that are important for you when there was a diagnosis of cancer or a worsening of your cancer or a complication from cancer.***

During the interview I will ask you some questions about your thoughts on your experience while you/your family member were in hospital and highlight those that were most and least helpful in goals of care discussions. There are no right or wrong answers to any of our questions, we are interested in your own experiences.

Participation in this study is voluntary and your decision to participate, or not participate, will not affect the care you or your family member receives. The interview should take between thirty minutes to one hour depending on how much information you would like to share. With your permission, I would like to audio record the interview because I don’t want to miss any of your comments. All responses will be kept confidential. This means that your de-identified interview responses will only be shared with research team members and we will ensure that any information we include in our report does not identify you as the respondent. You may decline to answer any question or stop the interview at any time and for any reason.

Are there any questions about what I have just explained?

May I start recording?

*Please note that this guide only represents the main themes to be discussed with the participants and as such does not include the various prompts that may also be used (examples given for each question). Non-leading and general prompts will also be used, such as “Can you please tell me a little bit more about that?” and “What does that look like for you”.*

| Establishing Rapport | ***Before we begin, it would be nice if you could tell me a little bit about yourself/your family member. Tailor a question here to specific person and/or situation.*** |
| --- | --- |
| General Description of Serious Illness Conversations | ***Serious illness is defined as a health condition that carries a high risk of mortality and either affects a person’s quality of life or function negatively or provides excessive demands on caregivers. Generally, how has your experience with serious illness conversations been with health care providers when you or your family member have been in hospital?***  Prompt: If you recall, who in the health-care team had this conversation with you/your family member?  Prompt: Did you think the timing of this conversation was a good timing? Why or why not?  Prompt: How would you describe your/your family member’s relationship with the health care provider discussing the serious illness conversation? Were you comfortable having this conversation?  Prompt: How did you/your family member feel after this conversation? |
| Communication Barriers | ***Can you recall any communication barriers you/your family member faced with health care providers while having these serious illness conversations?***  Prompt: Did you/your family member understand what was being said? Did the health care provider(s) use terminology/medical jargon you/they didn’t understand?  Prompt: Did you/your family member feel comfortable asking questions? Were questions answered?  Prompt: Is there anything you/your family member would have preferred the health care provider did differently? |
| Resources, Collaboration and Support | ***Did you/your family member receive any resources or support during this serious illness conversation?***  ***Did you feel that all members of your care team understood your wishes?***  Prompt: If no, would you/your family member have appreciated receiving something?  Prompt: Was there a missed opportunity to bring in other members of your care team for these conversations?  Prompt: If yes, were these resources/support sufficient?  Prompt: Is there anything you/your family member or another member of your care team may have wished further information about? |
| Role of Caregiver | ***Patients: Did you have anyone with you (e.g., spouse, children, friend, etc.) when you were involved in a serious illness conversation with a health care provider?***  Prompt: If not, why? Would you have liked them to be involved and if so, why? How?  Prompt: If yes, did it help to have them there?  ***Caregivers: Were you present when your family member was involved in a serious illness conversation with a health care provider?***  Prompt: If not, why? Would you have liked to be involved and if so, why? How?  Prompt: If yes, was it beneficial to your family member to have you there? |
| Role of Determinants of Health | ***We have heard from patients we have previously interviewed*** ***that various factors such as gender, language, culture, religion etc. can affect one’s experience with serious illness conversations.***    Prompts: Are there any factors that affected your/your family member’s experience with serious illness conversations? |
| Conclusion | ***Given the nature of what we have discussed, is there anything else that you would like to comment on or share?*** |

*Thank you very much for your time and the information you shared today.*
